# Research of personality anxiety and anxiety disorders in patients receiving opioid agonist maintenance therapy (OAMT)

**DOI:** 10.1192/j.eurpsy.2025.951

**Published:** 2025-08-26

**Authors:** N. Halytska-Pasichnyk

**Affiliations:** OAMT, Municipal Enterprise “Dnipropetrovsk multiprofile clinical hospital for provision of psychiatric care” Dnipropetrovsk regional council”, Dnipro, Ukraine

## Abstract

**Introduction:**

Increased levels of anxiety in patients with opioid dependence is a common problem that requires a comprehensive approach to treatment. They can manifest as GAD, panic attacks, social anxiety, and other forms of anxiety. The level of personal distress in patients on methadone therapy can be significant, as these patients often face a complex of psychological and social problems related to addiction and treatment.

**Objectives:**

The causes of anxiety disorders can be: 1) biological factors - changes in the neurochemistry of the brain associated with the use of opioids, which can affect the regulation of emotions and cause anxiety; 2) psychological factors – trauma in the anamnesis, stressful situations or negative experiences can contribute to the development of anxiety disorders; 3) social factors – relationship problems, social isolation and economic hardship can increase anxiety.

Factors affecting the level of personal anxiety include both medical and social aspects - a) changes in life; b) concomitant mental disorders; c) attitude toward therapy; d) social support.

**Methods:**

In the course of the study, 150 patients aged 26 to 64 years with a diagnosis of opioid dependence, who receive methadone hydrochloride as OAMT, were examined. The Psychopathological Symptom Severity Questionnaire (SCL-90-R, Derogatis, Lipman, Covi) and the Hamilton Anxiety Rating Scale (HAM-A/HARS) were used to assess the level of personal anxiety and existing anxiety disorders.

**Results:**

According to the levels of interpersonal anxiety (very low, low, medium, high), the indicators were distributed as follows: in the control group - 10 (20.4%), 32 (65.3%), 7 (14.3%), 0 (0 .0%); in the main group - 28 (27.7%), 59 (58.4%), 13 (12.9%), 1 (1.0%), respectively (p=0.685).

The degree of clinical anxiety according to the Hamilton scale (mild, moderate, severe, strong) in the main group was distributed as follows: 60 (59.41%), 23 (22.77%), 15 (14.85%) and 3 (2.97%); in the control group - 31 (63.27%), 10 (20.41%), 6 (12.24%), 2 (4.08%), respectively (p=0.930).

The level of anxiety according to the SCL-90 scale (very low, low, medium, elevated, high) was determined in 19 (38.78%), 22 (44.90%), 6 (12.24%), 2 (4.08%) and 0 (0.00 %) of persons, respectively, in the control group; 39 (38.61%), 46 (45.54%), 15 (14.85%), 1 (0.99%) and 0 (0.00%) - in the primary (p=0.628).

**Conclusions:**

The results of the study showed that receiving substitution maintenance therapy did not significantly increase the level of personal anxiety and the risk of anxiety disorders in patients on methadone therapy, the vast majority of patients showed very low, low and mild levels of manifestations. However, the level of personal anxiety may vary, but it is important to provide a comprehensive approach to treatment, including psychological support and social integration, to improve patients’ quality of life and reduce anxiety.

**Disclosure of Interest:**

None Declared

